# Methylene-Blue-Encapsulated Metal-Organic-Framework-Based Electrochemical POCT Platform for Multiple Detection of Heavy Metal Ions in Milk

**DOI:** 10.3390/bios13080783

**Published:** 2023-08-02

**Authors:** Huiwen Xiong, Pintao Li, Fei Cun, Hui Chen, Jilie Kong

**Affiliations:** Department of Chemistry, Fudan University, Shanghai 200438, China

**Keywords:** heavy metal ions, point-of-care testing, electrochemical detection, metal organic frameworks, on-site

## Abstract

Considering the high risk of heavy metal ions (HMIs) transferring through the food chain and accumulating in milk, a flexible and facile point-of-care testing (POCT) platform is urgently needed for the accurate, sensitive, and highly selective on-site quantification of multiple HMIs in milk. In this work, a cost-effective disk with six screen-printed electrodes (SPEs) was designed for hand-held electrochemical detection. Metal organic frameworks (MOFs) were adopted to amplify and enhance the electrochemical signals of methylene blue (MB). Using differential pulse voltammetry (DPV) methods, low limits of detection for four HMIs (Cd^2+^, 0.039 ppb; Hg^2+^, 0.039 ppb; Pb^2+^, 0.073 ppb; and As^3+^, 0.022 ppb) were achieved within four minutes. Moreover, the quantitative POCT system was applied to milk samples. The advantages of low cost, ease of on-site implementation, fast response, and accuracy allow for the POCT platform to be used in practical monitoring applications for the quantitation of multiple HMIs in milk samples.

## 1. Introduction

With the improvements in quality of life and the development of industrialization, the risk of contaminating nutritious milk products has gradually gained attention. In particular, heavy metal ions (HMIs) in milk have become a significant problem for food safety [1]. HMIs often exist in raw milk and milk powder, which may enter the human body through ingestion and cause considerable harm owing to recalcitrance, high toxicity, and facile enrichment [2,3]. The maximum permitted concentrations of Hg^2+^, Pb^2+^, and As^3+^ in raw milk are limited to 0.01, 0.05, and 0.1 mg/kg by the National Standard of the People’s Republic of China (GB 2762-2012), respectively. Conventional detection methods for HMIs mainly include atomic absorption spectrometry (AAS) [4], inductively coupled plasma mass spectrometry (ICP-MS) [5], atomic fluorescence spectrometry (AFS) [6], surface-enhanced Raman spectroscopy (SERS) [7,8], immunoassays [9], and fluorescence (FL) methods [10]. However, these techniques rely on expensive instruments and are too sophisticated to support rapid point-of-care testing (POCT). Hence, HMI sensing devices that are portable, simple to use, and cost-effective must be developed.

POCT allows non-specialists to quickly perform on-site analysis, using low and disposable materials compared with tests in laboratories [11]. POCT devices, such as microfluidic platforms [12,13] and paper-based sensors [2], have the advantages of facile operation, low material consumption, rapid output, and versatile application with existing infrastructure [14]. Therefore, POCT devices have been adopted in food safety analysis [15], clinical diagnosis [16], and environmental monitoring [17].

Integrated electrochemical POCT sensing devices have received attention because of their small size and high sensitivity [18]. Furthermore, the electrochemical POCT devices produce direct electrical signals based on the oxidation–reduction reaction. Therefore, it is easy to integrate them with signal recording and processing components in compact analytical devices [19,20]. Various electrochemical techniques, such as cyclic voltammetry (CV) [21], differential pulse voltammetry (DPV) [22], and electrochemical impedance spectroscopy (EIS) [23], have been applied in the field of smartphone-based POCT for the quantitative detection of HMIs. Among them, DPV has been commonly adopted owing to the high detection accuracy and reduction in background current. For instance, Xu et al. presented an electrochemical POCT platform for the simultaneous quantitative detection of four HMIs (Cd^2+^, Hg^2+^, Pb^2+^, and Cu^2+^) with adequate limits of detection (LODs) (0.296, 0.351, 0.025, and 0.055 μM, respectively) by employing reduced graphene oxide to accelerate electron transfer [22]. However, the detection of four HMIs was performed simultaneously on the same electrode, which might contribute to the lower sensitivity and higher interferences of each HMI. Moreover, the electrochemical signals mainly depend on self-oxidation and reduction, which may be undetected at very low concentrations of each HMI, thus limiting the sensitivity of the POCT platform. Therefore, it is worthwhile to develop a more effective sensing strategy.

Aptamers, which are small single-stranded nucleic acids or peptide molecules selected via the systematic evolution of ligands using exponential enrichment (SELEX) technology, can bind with target molecules [24,25]. Recently, aptamer-based sensors have been considered in environmental monitoring [26], bioanalysis [27], and food safety [28]. Compared with antibodies, aptamers have higher selectivity and affinity, higher stability, and lower cost [29]. Aptamers are commonly utilized for the binding strategy in electrochemical detection methods owing to their unique advantages of high sensitivity, quick output, and low detection limits [30]. Abu-Ali et al. fabricated an aptamer-based electrochemical biosensor for the detection of Hg^2+^ and Pb^2+^ ions utilizing ferrocene or methylene blue (MB) as the electroactive materials [31]. The electrochemical signal of MB increased after binding with the target HMIs because the conformational changes in the aptamers accelerated the electron transfer between MB and the electrode. However, the biosensor cannot support ultrasensitive electrochemical aptasensing in POCT because of the relatively small electrode surface areas. Therefore, improving the loading of electroactive materials is necessary to increase the sensitivity of electrochemical biosensors.

Metal–organic frameworks (MOFs) consist of metal centers and organic ligands [32,33,34]. Owing to the unique features of high surface areas and variable porosity, MOFs have become promising materials in the field of sensing, adsorption, and biomedical applications [32,35,36,37,38]. For the detection of HMIs, MOFs have been employed to provide binding sites because of their large surface areas based on organic ligands [22,39]. Thus, MOFs are considered effective materials to load electroactive materials and improve the sensitivity of sensors [40]. The well-defined porosity of MOFs accommodates high concentrations of electroactive materials, such as MB and Ru(bpy)_3_^2+^, thus increasing the conductivity and sensitivity of the electrochemical sensor [41,42]. Among them, the UiO-66-NH_2_ MOF based on Zr (IV) exhibits adjustable nanoporosity, electrochemical stability, abundant surface amino groups, and high encapsulation efficiency [43]. Moreover, Zr-MOFs can be easily modified with DNA strands owing to the Zr-P bond [44]. Therefore, Zr-MOFs were chosen in the present study, as an ideal loading material for biochemical assay probes. To date, MOFs have not been used for encapsulating electroactive molecules in aptamer-based electrochemical POCT platforms.

Herein, we present an electrochemical POCT aptasensing platform for the rapid quantitative detection of Cd^2+^, Hg^2+^, Pb^2+^, and As^3+^. The platform contained a portable smartphone-driven electrochemical analyzer and six screen-printed electrodes (SPEs) on a polyethylene glycol terephthalate (PET) disk. We employed a Zr-MOFs as a carrier for loading high concentrations of MB to fabricate electrochemical nanoprobes (MB@Zr-MOFs). Gold nanoparticles (AuNPs) were used to link the modified aptamers firmly with the SPEs via the Au−S bond, and the high conductivity of the AuNPs supported efficient electron transfer on the SPEs. The cheap and disposable electrochemical POCT aptasensor was used to demonstrate the on-site analysis of Cd^2+^, Hg^2+^, Pb^2+^, and As^3+^ in milk with high sensitivity, thus advancing the development of POCT platforms.

## 2. Materials and Methods

### 2.1. Materials and Reagents

Standard solutions of Cd (II), Pb (II), Hg (II), and As (III) in 1.0 mol/L HNO_3_, chloroauric acid (HAuCl_4_·3H_2_O), trisodium citrate, ethanol, ZrCl_4_, MB, aminoterephthalic acid (NH_2_-BDC), tris (2-carboxyethyl) phosphine (TCEP), 6-mercapto-1-hexanol (MCH), and benzoic acid were purchased from Aladdin Chemistry Co., Ltd. (Shanghai, China). N, N dimethylformamide (DMF) was obtained from Sigma Aldrich (Shanghai, China), and 0.1 M phosphate buffered saline (PBS, pH 7.4) containing Na_2_HPO_4_ and KH_2_PO_4_ was obtained from Thermo Fisher Scientific (United States). The oligonucleotides with sequences of 5′-COOH-CTCAGGACGACGGGTTCACAGTCCGTTGTC-SH-3′ for Cd^2+^ [45], 5′-COOH-TTGTTTGTCCCCTCTTTCTTA-(CH_2_)_3_-SH-3′ for Hg^2+^ [46], 5′-COOH-CAACGGTTGGTGTGGTTGG-SH-3′ for Pb^2+^ [47], and 5′-COOH-GTGTGTGTGTGTGTGTGTGTGTGTGTGTGTGTGTGTGTGTGT-(CH_2_)_3_-SH-3′ for As^3+^ [48] were synthesized by Sangon Biotechnology Inc. (Shanghai, China). Ultrapure water (Milli-Q grade, Millipore, Burlington, MA, USA) at 25 °C with a resistivity of 18.2 MΩ·cm was used throughout the experiment. Milk deluxe was bought from the supermarket and stored at room temperature for further use.

### 2.2. Instrumentation

The disks containing six SPEs, a PET substrate, and a polydimethylsiloxane (PDMS) insulating loop were obtained from Weihai Poten Technology Co., Ltd. (Weihai, China). The six SPEs included four carbon electrodes as the working electrodes, Ag/AgCl as the reference electrode, and a carbon electrode as the counter electrode. DPV was performed using a BIOSYS p15e max biosensor system from Shenzhen Refresh Biosensing Technology Co., Ltd. (Shenzhen, China). This hand-held electrochemical station was driven by a smartphone installed with an application that uses Blue tooth. CV and EIS were conducted using a CHI660E electrochemical station (Chenhua, Shanghai, China). The morphologies and energy-dispersive X-ray (EDX) patterns of MOFs and MB@MOFs were obtained through high-resolution transmission electron microscopy (HRTEM) using an HT7700 Exalens microscope (Hitachi, Tokyo, Japan) at an accelerating voltage of 120 kV. X-ray diffraction (XRD) patterns were obtained using a D2 PHASER diffractometer (BRUKER, Mannheim, Germany). Adsorption–desorption isotherms were assessed using an automated surface area and porosity analyzer (Quantachrome, Boynton Beach, FL, USA).

### 2.3. Preparation of AuNPs

The synthesis of AuNPs was conducted using a citrate reduction method, in accordance with previous reports [49]. Briefly, 500 μL of 2% HAuCl_4_ aqueous solution and 100 mL of distilled water were mixed in a flask and boiled under constant stirring. Next, 500 μL of 1% sodium citrate was added and the mixture was stirred for an additional 20 min. The wine-red AuNP solution was cooled to room temperature and stored at 4 °C.

### 2.4. Preparation of Zr-MOFs

UiO-66-NH_2_ was synthesized using the procedure reported in a previous report with a slight modification [50]. First, 120 mg of ZrCl_4_, 1900 mg of benzoic acid, and 110 mg of 2-aminoterephthalic acid (NH_2_-BDC) were dissolved in 10 mL of DMF under ultrasonication for 3 min. The dispersion was then reacted at 120 °C for 24 h. Next, the precipitate was obtained after washing twice with DMF and methanol. Finally, the Zr-MOF precipitates were dried at 65 °C for 12 h and kept in a desiccator.

### 2.5. Preparation of MB@Zr-MOFs

First, 1 mg of MB was mixed with 10 mg of Zr-MOFs in 1 mL of distilled water using a rotary mixer at room temperature for 24 h [42]. Then, the precipitate was centrifuged and washed three times with ultrapure water. The resulting blue powder was dried at 65 °C overnight and stored in the dry environment for further use.

### 2.6. Preparation of MB@Zr-MOFs-apt and MB-apt

Here, 300 μL of 10 μM aptamers targeting different HMIs was activated with 5 μL of 1 M TCEP for 30 min at room temperature to convert the disulfide bonds to thiols. Then, 800 μL of 2.5 μM aptamers with carboxyl groups was mixed with 400 μL of EDC/NHS (400 mM/100 mM) in 10 mM PBS solution (pH 7.4) [51]. The mixture was stirred for 30 min to activate the aptamer carboxy groups. Next, the amide reaction was conducted with the addition of 800 μL of 1 mg/mL MB@Zr-MOFs for 2 h. The light blue precipitate was obtained after centrifugation at 12,000 rpm for 20 min. Finally, the MB@Zr-MOFs-apt complex was attained and redispersed in 800 μL of 10 mM PBS solution (pH 7.4). For preparation of the MB-apt, the steps were basically same as those for MB@Zr-MOFs-apt except for the addition of 800 μL of 1 mg/mL MB.

### 2.7. Fabrication of the Portable Electrochemical Platform

Prior to modification, the bare SPEs were cleaned in 0.5 M H_2_SO_4_ under CV from −0.5 to +1.5 V until reproducible voltammograms were achieved [52]. Then, the four working SPEs were modified using 2.5 μL of AuNPs and dried in air [51]. Each working SPE was incubated with 2.5 μL of MB@Zr-MOFs-apt for 8 h at 4 °C to form strong Au–S bonds and promote complete coverage. Then, the aptamer-modified SPEs were treated with 2.5 μL of 5.0 mM MCH for 1 h to block the unspecific binding sites [53]. Finally, the as-prepared aptasensor was thoroughly rinsed with 10 mM PBS (pH 7.4). To perform HMI detection using the electrochemical POCT aptasensing platform, the disk was completely immersed in 200 μL of PBS (pH 7.4) containing 10 ppb each of Cd^2+^, Hg^2+^, Pb^2+^, and As^3+^. The disk was incubated for 30 min and then thoroughly rinsed with ultrapure water and stored at 4 °C.

The electrochemical responses were measured and recorded based on the DPV method using PBS (pH 7.4) as the supporting electrolyte. The parameters of the DPV method were set as follows: amplitude potential = 0.1 V, pulse width = 0.1 s, pulse period = 1 s, quiet time = 2 s, and a potential range from −0.2 to +0.5 V. Each experiment was carried out three times, and results were recorded as the mean ± standard deviation (SD).

## 3. Results and Discussion

### 3.1. Detection Principle of the Electrochemical POCT Platform

**Scheme 1:**
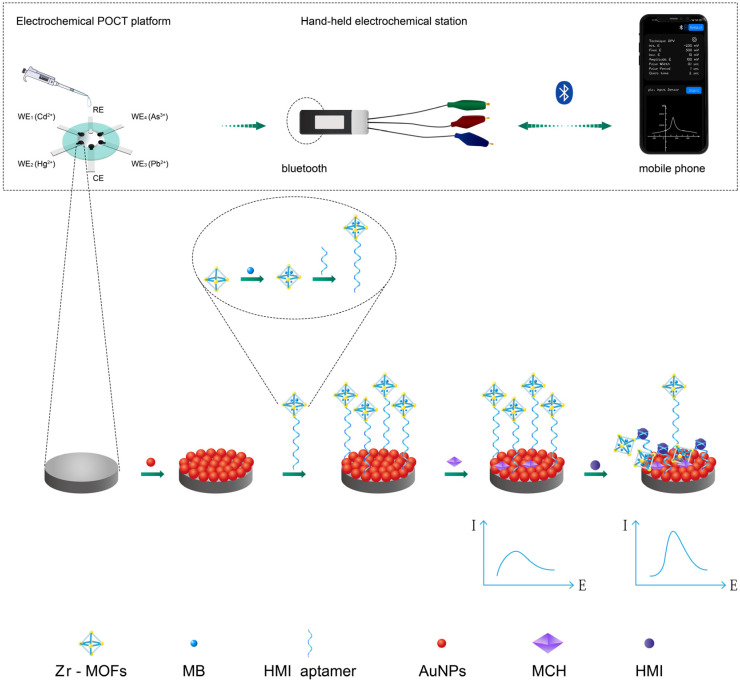
Principle of the electrochemical POCT aptasensing platform for detection of four HMIs.

A photograph of the portable electrochemical workstation and electrochemical POCT platform is provided in Figure S1. As shown in Scheme 1, the electrochemical POCT system consisted of a PET disk with a PDMS insulation loop and a portable electrochemical station. The outer diameter of the disk was 2 cm (almost a size of a coin), and the inner diameter was 1.2 cm. The diameters of the working SPEs were 1.8 mm. Furthermore, we designed the platform with six SPEs in specific positions, to simplify the operation complexity. For example, the reference electrode was placed opposite the counter electrode, with the same diameters, to avoid spilling of the PBS reaction solution and short circuiting of the conducting wires. In advance, aptamers modified with carboxyl groups were linked covalently with MB@Zr-MOFs modified with amino groups via amide reactions. AuNPs were used on all four working SPEs to provide effective electron transfer and serve as the loading substrates to support the thiolated aptamers. Then, the MB@Zr-MOFs-apts were immobilized on the surfaces of the AuNPs via the Au–S bond, and MCH was added to block the non-specific binding sites. The relatively weak DPV responses were observed owing to the resistive conformation of the electroactive MB@Zr-MOFs on the SPE surfaces. The target HMIs, were recognized by the corresponding specific aptamers upon addition and captured on the SPE surfaces, leading to a change in the conformation and the enhanced proximity of electroactive MB@Zr-MOFs with the electrode surface. Therefore, the DPV signals of the aptasensors were greatly enhanced. The DPV current increased with an increasing number of HMIs (*Δ* is the difference in the presence and absence of the four HMIs). The whole test lasted approximately 4 min: 2 min for DPV detection of the four HMIs and 2 min for changing PBS solutions and four conducting WEs. Thus, the proposed electrochemical POCT system enabled the convenient on-site detection of four HMIs.

### 3.2. Characterization of Zr-MOFs and MB@Zr-MOFs

**Figure 1:**
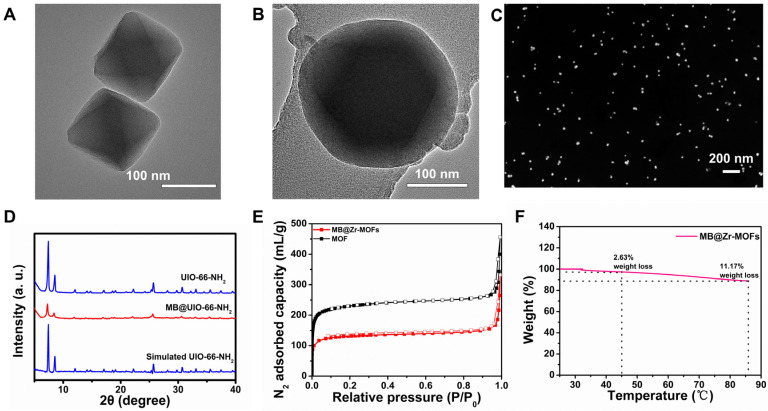
HRTEM images of (**A**) Zr-MOFs crystals, (**B**) the nanocomposites MB@Zr-MOFs. (**C**) SEM images of AuNPs. (**D**) XRD patterns of simulated Zr-MOFs, as-synthesized Zr-MOFs crystals, and MB@Zr-MOFs. (**E**) Nitrogen adsorption–desorption isotherm of Zr-MOFs and MB@Zr-MOFs. (**F**) TGA diagram of MB@Zr-MOFs.

The morphologies of Zr-MOFs and MB@Zr-MOFs were characterized using HRTEM. The Zr-MOF is a light-yellow powder (Figure S2B) with a uniform particle shape and size of 180 nm (Figure 1A). After MB was loaded into the Zr-MOFs, the size slightly increased to 200 nm, and the shape became round, demonstrating that loading with the small molecules has little influence on the size (Figure 1B). The loading of MB on Zr-MOFs was confirmed through EDX mapping (Figures S3 and S4), which revealed an increase in the sulfur concentration after loading. Moreover, the scanning electron microscopy (SEM) of the AuNPs revealed their homogeneous dispersion with a diameter of 20 nm on the SPEs (Figure 1C). As shown in Figure 1D, the XRD patterns of simulated Zr-MOFs, Zr-MOFs, and MB@Zr-MOFs showed almost the same diffraction peak positions, indicating that MB had little impact on the crystalline structure of Zr-MOFs. Adsorption–desorption isotherms were obtained at 77.3 K to verify the synthesis of MB@Zr-MOFs [54]. As shown in Figure 1E, the Brunauer−Emmett−Teller (BET) surface area of Zr-MOFs was calculated as 814 m^2^/g, whereas the BET surface area decreased to 465 m^2^/g when the electroactive dyes occupied the pores of the Zr-MOFs, demonstrating the successful encapsulation of MB in Zr-MOFs. The loading efficiency of MB in Zr-MOFs was estimated as (814 − 465)/814×100% = 42.9%. The morphologies and adsorption characteristics were comparable with those reported in previous work [42]. In addition, the sensing stability of MB@Zr-MOFs was inspected based on the TGA curve under an oxygen atmosphere. As shown in Figure 1F, no weight loss was observed before 32 °C. Moreover, the weight loss was lower than 5 wt% before 45 °C, which clearly illustrated that MB@Zr-MOFs were kept stable under the sensing environment at room temperature.

### 3.3. Amplification Electrochemical Behavior of the POCT Sensor

**Figure 2:**
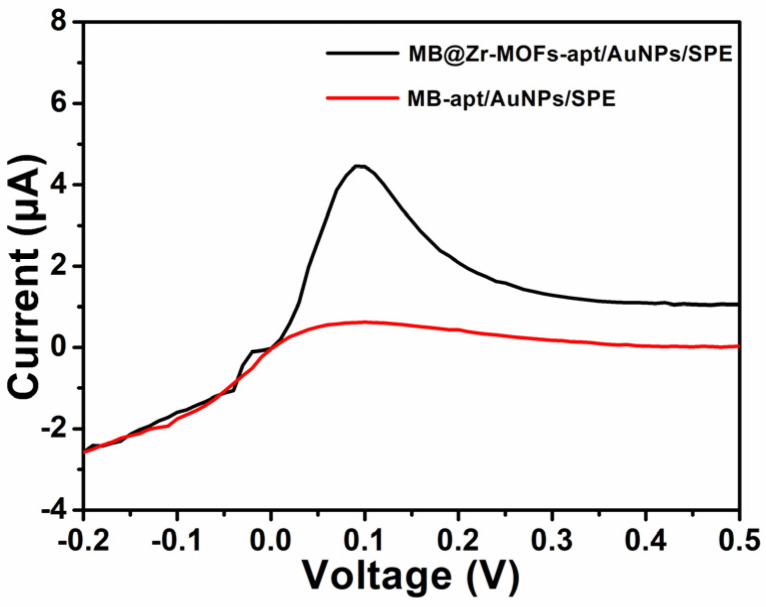
DPV response of the MB@Zr-MOFs-apt/AuNPs/SPE (black curve) and MB-apt/AuNPs/SPE (red curve). All DPV experiments were carried out using 0.1 M PBS (pH 7.4) solution at a potential of −0.2 to +0.5 V.

To explore the electrochemical amplification effect of Zr-MOFs, DPV signals of MB were tested with and without being loaded into Zr-MOFs. The bare SPEs were cleaned and modified using the same protocol of the established platform, except for incubation with 2.5 μL of MB-apt for 8 h at 4 °C to form tight Au–S bond. The final concentration of apt in MB-apt was the same with that of 2.5 μM in MB@Zr-MOFs-apt. As shown in Figure 2, the DPV peak of MB-apt/AuNPs/SPE (red curve) was located at approximately +0.10 V, and the current was 0.623 μA. In contrast, the DPV peak of MB@Zr-MOFs-apt/AuNPs/SPE was located at approximately +0.09 V and the current was 4.46 μA, increasing by 7.2 times compared with that of the red curve. The results suggest that loading MB into Zr-MOFs can greatly enhance the DPV signals of MB.

### 3.4. Electrochemical Behavior of the POCT Sensor

**Figure 3:**
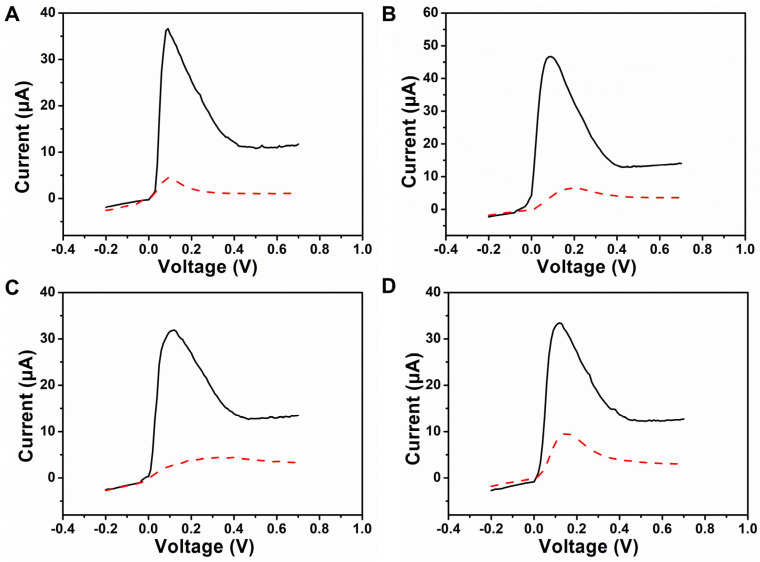
DPV response of the electrochemical POCT aptasensors to 10 ppb (**A**) Cd^2+^, (**B**) Hg^2+^, (**C**) Pb^2+^, and (**D**) As^3+^. The dotted line is MCH/MB@Zr-MOFs-apt/AuNPs/SPE, and the solid line is HMIs/MCH/MB@Zr-MOFs-apt/AuNPs/SPE. All DPV experiments were carried out using 0.1 M PBS (pH 7.4) solution at a potential that varied from −0.2 to +0.5 V.

The electrochemical behavior of four HMIs was investigated using DPV. The SPEs on the disk with different aptamer modifications were tested in PBS (pH 7.4) solution. As shown in Figure 3, slight DPV peak currents located at approximately +0.11 V were observed for the four SPEs with the addition of MCH, suggesting that the aptasensors were slightly electroactive in the potential windows. The peak currents increased after the disk was immersed in PBS (pH 7.4) solution containing 10 ppb each of Cd^2+^, Hg^2+^, Pb^2+^, and As^3+^ (Figure 3A for Cd^2+^, Figure 3B for Hg^2+^, Figure 3C for Pb^2+^ and Figure 3D for As^3+^). These results demonstrated that the conformational changes of the aptamers that captured the HMIs could shorten the distance between the electroactive MB@Zr-MOFs and the electrode surface [31].

### 3.5. Analytical Performance of the Electrochemical POCT Sensor

**Figure 4:**
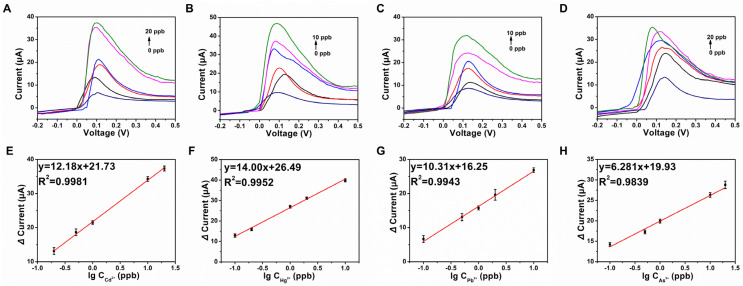
DPV responses of (**A**) Cd^2+^ at concentrations of 0, 0.2, 0.5, 1.0, 10, and 20 ppb; (**B**) Hg^2+^ at concentrations of 0, 0.1, 0.2, 1.0, 5.0, and 10 ppb; (**C**) Pb^2+^ at concentrations of 0, 0.1, 0.5, 1.0, 2.0, and 10 ppb; and (**D**) As^3+^ at concentrations of 0, 0.1, 0.5, 1.0, 10, and 20 ppb. Calibration curves of (**E**) Cd^2+^, (**F**) Hg^2+^, (**G**) Pb^2+^, and (**H**) As^3+^ using the electrochemical POCT device. The experiment was carried out in 0.1 M PBS (pH 7.4) at a potential of −0.2 and 0.5 V. Lines in different color represent different concentrations of HMIs.

Under optimized conditions using 6 μM aptamer concentrations and 45 min of incubation time for HMIs (Figure S10A,B), DPV was performed to determine the analytical performance of the proposed aptasensor for HMI detection. The results for different HMI concentrations are shown in Figure 4. As shown in Figure 4A–D, the DPV current increased linearly with the increase in each HMI concentration. The aptasensing platform exhibited useful analytical performance, with a dynamic range of 0.2–20 ppb for Cd^2+^, 0.1–10 ppb for Hg^2+^, 0.1–10 ppb for Pb^2+^, and 0.1–20 ppb for As^3+^.

As shown in Figure 4E–H, a series of different standard concentrations of HMIs was applied to estimate the analytical performance. Utilizing a series of standard concentrations of Cd^2+^, Hg^2+^, Pb^2+^, and As^3+^, the current difference of aptasensor signals in the presence and absence of each HMI was defined as the *Δ* current. The DPV *Δ* current responses exhibited linearity as the concentration increased from a concentration of 0.2 to 20 ppb for Cd^2+^, 0.1 to 10 ppb for Hg^2+^, 0.1 to 10 ppb for Pb^2+^, and 0.1 to 20 ppb for As^3+^. Four calibration graphs were observed with the linear regression equations *Δ* current (μA) = 12.18 lg C_Cd(II)_ + 21.73 (R^2^ = 0.9981), *Δ* current (μA) = 14.00 lg C_Hg(II)_ + 26.49 (R^2^ = 0.9952), *Δ* current (μA) = 10.31 lg C_Pb(II)_ + 16.25 (R^2^ = 0.9943), and *Δ* current (μA) = 6.281 lg C_As(III)_ + 19.93 (R^2^ = 0.9839). The LOD was calculated using 3σ/S, where σ is the SD of blank signals (n = 3), and S represents the slope of the calibration curves [55]. The LODs were 0.039 ppb for Cd^2+^, 0.039 ppb for Hg^2+^, 0.073 ppb for Pb^2+^, and 0.022 ppb for As^3+^. Notably, the LODs obtained using the disk aptasensing platform were lower than the national standard values [56,57], indicating that the constructed electrochemical POCT system is suitable and practical for the quantitative on-site detection of Cd^2+^, Hg^2+^, Pb^2+^, and As^3+^. Compared with previously reported aptasensors for Cd^2+^, Hg^2+^, Pb^2+^, and As^3+^ analysis, the proposed electrochemical POCT aptasensing platform achieved lower LODs and a relatively wide linear range (Table S1), indicating that the fabricated aptasensing platform was acceptable and has potential for real sample analysis. The electrochemical POCT platform exhibited good performance when compared to some previously reported methods for the detection of HMIs (Table S1).

### 3.6. Stability, Reproducibility, and Selectivity of Electrochemical POCT Platform

**Figure 5:**
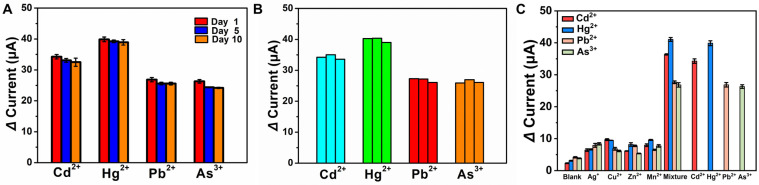
(**A**) Stability of the electrochemical POCT aptasensing platform for Cd^2+^, Hg^2+^, Pb^2+^, and As^3+^ (each at concentrations of 10 ppb). (**B**) Reproducibility of three electrochemical POCT aptasensing platforms. Each platform was tested under the same experimental conditions. The concentration of Cd^2+^, Hg^2+^, Pb^2+^, and As^3+^ was 10 ppb each. (**C**) The DPV response of the electrochemical POCT platform to different samples in PBS (0.1 M, pH 7.4): (a) blank (without any HMI), (b) Ag^+^, (c) Cu^2+^, (d) Zn^2+^, (e) Mn^2+^, (f) a mixture containing of 30 ppb of Ag^+^, Cu^2+^, Zn^2+^, Mn^2+^ and 10 ppb of Cd^2+^, Hg^2+^, Pb^2+^, and As^3+^.

It is essential for a POCT aptasensing platform to have good stability. The aptasensors were prepared using the same procedure and stored at 4 °C for further use. As shown in Figure 5A, the aptasensors retained 96.5% for Cd^2+^, 98.4% for Hg^2+^, 95.3% for Pb^2+^, and 92.7% for As^3+^of its initial electrochemical response after five days. Moreover, it retained 94.8% for Cd^2+^, 97.6% for Hg^2+^, 95.3% for Pb^2+^, and 91.9% for As^3+^of its initial electrochemical response after ten days. Therefore, the aptasensors remained bioactive within 10 days. The obtained results demonstrated that the proposed aptasensors have good storage and utilization stability.

For the reproducibility test, as shown in Figure 5B, the relative standard deviations (RSDs) for three parallel tests using HMI aptasensors under the same experimental conditions were 1.7% for Cd^2+^, 1.5% for Hg^2+^, 2.1% for Pb^2+^, and 1.8% for As^3+^. The results demonstrated their acceptable reproducibility.

The selectivity of the constructed electrochemical POCT aptasensing platform toward Cd^2+^, Hg^2+^, Pb^2+^, and As^3+^ was investigated by including other metal ions, including Ag^+^, Cu^2+^, Zn^2+^, and Mn^2+^. Specifically, the mixture solution contained 10 ppb each of Cd^2+^, Hg^2+^, Pb^2+^, and As^3+^ and 30 ppb each of Ag^+^, Cu^2+^, Zn^2+^, and Mn^2+^. As indicated in Figure 5C, the aptasensor displayed a negligible current signal difference in the presence and absence of the additional metal ions, even with concentrations that were 3-fold higher than those of Cd^2+^, Hg^2+^, Pb^2+^, and As^3+^. When the mixture of target HMIs and additional metal ions was incubated on the four SPEs, a slight change in the DPV response was observed compared with that of the standard HMI solutions on the corresponding SPEs. The mixture solution exhibited almost the same DPV signal differences as those observed independently in the standard HMI solutions (relative errors: 6.12%, 2.96%, 2.98%, and 1.97% for detection of Cd^2+^, Hg^2+^, Pb^2+^, and As^3+^, respectively). The obtained results demonstrated that the proposed electrochemical POCT aptasensing platform is highly selective for the detection of Cd^2+^, Hg^2+^, Pb^2+^, and As^3+^ under competitive HMI conditions.

### 3.7. Real Sample Analysis Using Milk

To assess the suitability of the proposed aptasensor for HMI analytical applications using real milk samples, 5 mL solutions of raw milk samples were preprocessed by adding 0.1, 1, and 10 ppb each of the four HMIs. To remove proteins from milk that are easily adsorbed or cause the reduction of HMIs, 2 mL of trichloroacetic acid was added and stirred on a vortex shaker for 5 min. The mixed solutions were centrifuged at 10,000× *g* for 15 min, and the supernatant was collected and filtered through a 0.22 μm membrane filter. To prevent the interference of the residual trichloroacetic acid, the DPV curves of negative controls with milk pretreated in the same way without the HMIs were measured. No electrochemical signals were observed within the voltage window, indicating that there was no or very few HMIs detected. Then, the samples were prepared by adding different concentrations (10 and 30 μM) of HMI solutions into the commercial milk.

As shown in Table 1, the recoveries of four HMIs were calculated as 92.0–105% for Cd^2+^, 97.8–108% for Hg^2+^, 96.0–102% for Pb^2+^, and 95.3–108% for As^3+^. Therefore, the proposed electrochemical POCT aptasensing platform can accurately detect four HMIs in raw milk samples.

## 4. Conclusions

In this work, we constructed a sensitive, accurate, and flexible POCT aptasensing platform for the on-site detection of Cd^2+^, Hg^2+^, Pb^2+^, and As^3+^ in milk samples. Furthermore, the proposed disk-like aptasensing platform was portable, cost-effective, and user-friendly owing to the highly efficient data collection enabled by the smartphone application and Bluetooth connection. Moreover, Zr-MOFs enabled considerable loading of the electroactive MB molecule for signal amplification. Under the optimal conditions, the platform exhibited a high sensitivity for the four target HMIs, with LODs as low as 0.039 ppb for Cd^2+^, 0.039 ppb for Hg^2+^, 0.073 ppb for Pb^2+^, and 0.022 ppb for As^3+^ (S/N = 3). Additionally, the individual target HMIs can be specifically detected in a mixture of HMIs and kept active for ten days, demonstrating the useful specificity and stability of the sensing platform. Overall, the integrated electrochemical POCT system can be extended for practical on-site applications in milk sample monitoring. Further studies can focus on developing more versatile multi-channel POCT sensors for the simultaneous detection of more HMIs.

## Figures and Tables

**Table 1:** Detection of HMIs in milk samples using the electrochemical POCT platform.

Sample	Added (ppb)				Found (ppb)				Recovery (%)			
	Cd^2+^	Hg^2+^	Pb^2+^	As^3+^	Cd^2+^	Hg^2+^	Pb^2+^	As^3+^	Cd^2+^	Hg^2+^	Pb^2+^	As^3+^
Milk 1	0	0	0	0	0	0	0	0	0	0	0	0
Milk 2	0	0	0	0	0	0	0	0	0	0	0	0
Milk 3	0	0	0	0	0	0	0	0	0	0	0	0
Milk 1	0.100	0.100	0.100	0.100	0.0920	0.104	0.096	0.106	92.0	104	96.0	106
Milk 2	1.00	1.00	1.00	1.00	1.04	1.08	1.02	1.08	104	108	102	108
Milk 3	10.0	10.0	10.0	10.0	10.5	9.78	9.86	9.53	105	97.8	98.6	95.3

## Data Availability

Not applicable.

## Supplementary Materials

The following supporting information can be downloaded at: https://www.mdpi.com/article/10.3390/bios13080783/s1, Figure S1: Photograph of (A) portable electrochemical workstation; (B) electrochemical POCT platform; Figure S2: Photograph of (A) MB; (B) Zr-MOFs; (C) MB@Zr-MOFs; Figure S3: EDX mapping of different elements (A) Zr; (B) C; (C) O; (D) N in Zr-MOF; Figure S4: EDX mapping of different elements (A) Zr; (B) C; (C) O; (D) N; (E) in MB@Zr-MOFs nanocomposite; Figure S5: Cyclic voltammograms obtained by modified Pb^2+^-aptasensor in 0.1 M PBS (pH 7.4) solution; Figure S6: Powder X-ray diffraction patterns of MB@Zr-MOFs samples measured for chemical stability tests: as-synthesized sample and that soaked in 0.1 M PBS (pH 7.4) for up to 24 h; Figure S7: FT-IR spectroscopy of MB, Zr-MOFs and MB@Zr-MOFs; Figure S8: (A) CV and (B) EIS of the stepwise modifying validation of SPEs: (a) bare SPE, (b) AuNPs/SPE, (c) MB@Zr-MOFs-apt/AuNPs/SPE, (d) MCH/MB@Zr-MOFs-apt/AuNPs/SPE, (e) Cd^2+^/MCH/MB@Zr-MOFs-apt/AuNPs/SPE. The concentration of Cd^2+^ is 10 ppb; Figure S9: DPV curves of MCH/aptCd-MB@MOF/AuNPs/carbon rod; Figure S10: Optimizations of the (A) concentration of aptamers targeting Cd^2+^, Hg^2+^, Pb^2+^, As^3+^ (B) incubation time of Cd^2+^, Hg^2+^, Pb^2+^, As^3+^; Table S1: Comparison of analytical performance of POCT electrochemical platform for detection of HMIs with the previous reported HMIs detection methods. References [58,59,60,61,62] are cited in the supplementary materials.
