# Supplementary material for: Methylene-Blue-Encapsulated Metal-Organic-Framework-Based Electrochemical POCT Platform for Multiple Detection of Heavy Metal Ions in Milk
Source: Biosensors (Basel). 2023 Aug 2;13(8):783. doi: 10.3390/bios13080783 (PMC10452309; doi:10.3390/bios13080783)
Supplement: Supplementary file 1 [file biosensors-13-00783-s001.zip › biosensors-2513070-supplementary.pdf]

Supplementary Material

Methylene-Blue-Encapsulated Metal-Organic-Framework-Based Electrochemical POCT Platform for Multiple Detection of Heavy Metal Ions in Milk

Huiwen Xiong [a], Pintao Li [a], Fei Cun [a], Hui Chen [a, *], and Jilie Kong [a, *]

[a] Department of Chemistry, Fudan University, Shanghai 200438, China

***Corresponding Author**

* Hui Chen, chenhui@fudan.edu.cn;

* Jilie Kong, jlkong@fudan.edu.cn;

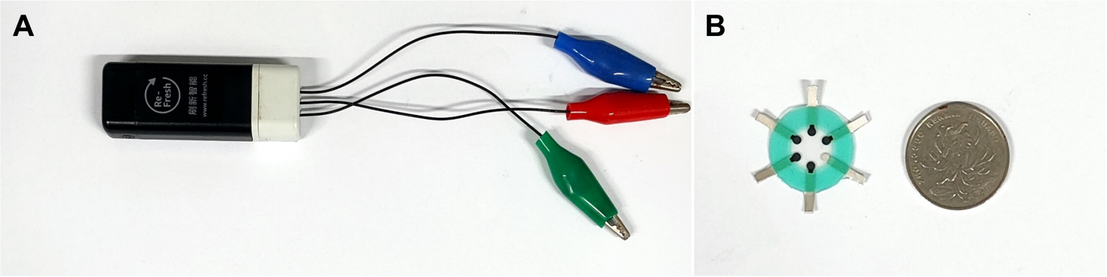

Figure S1. Photograph of (A) portable electrochemical workstation; (B) electrochemical POCT platform.

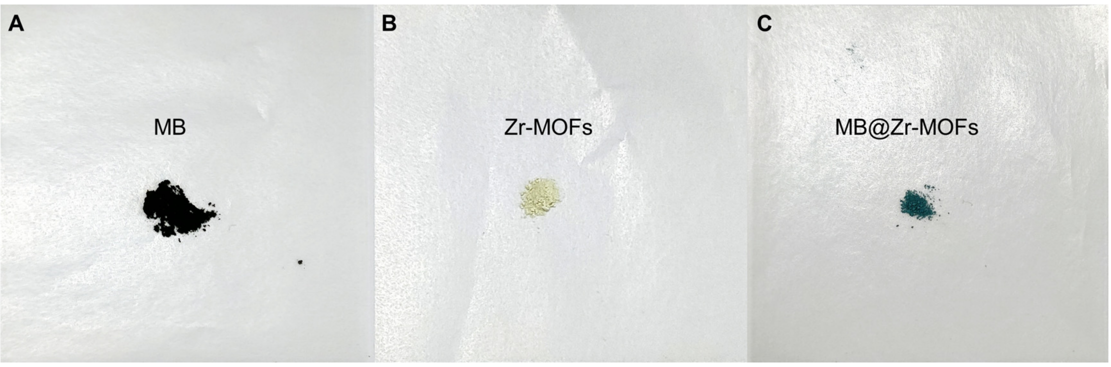

Figure S2. Photograph of (A) MB; (B) Zr-MOFs; (C) MB@Zr-MOFs.

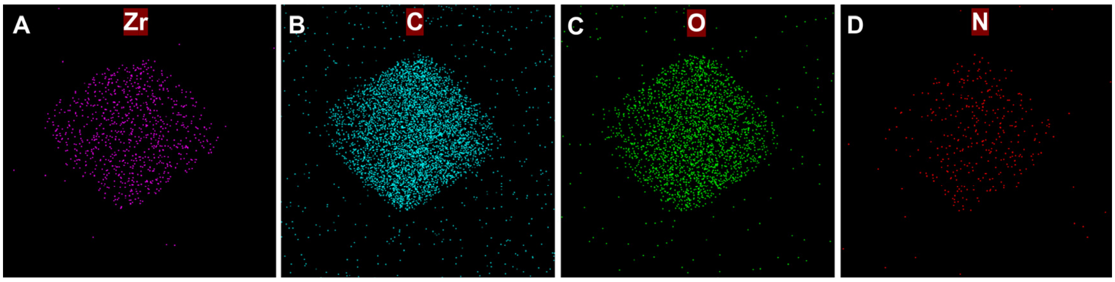

Figure S3. EDX mapping of different elements (A) Zr; (B) C; (C) O; (D) N in Zr-MOF.

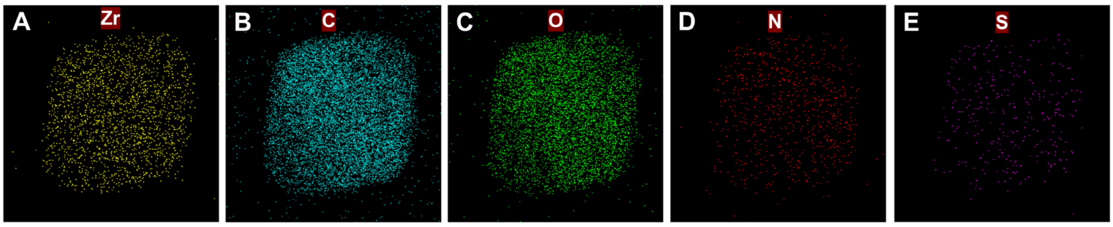

Figure S4. EDX mapping of different elements (A) Zr; (B) C; (C) O; (D) N; (E) in MB@Zr-MOFs nanocomposite.

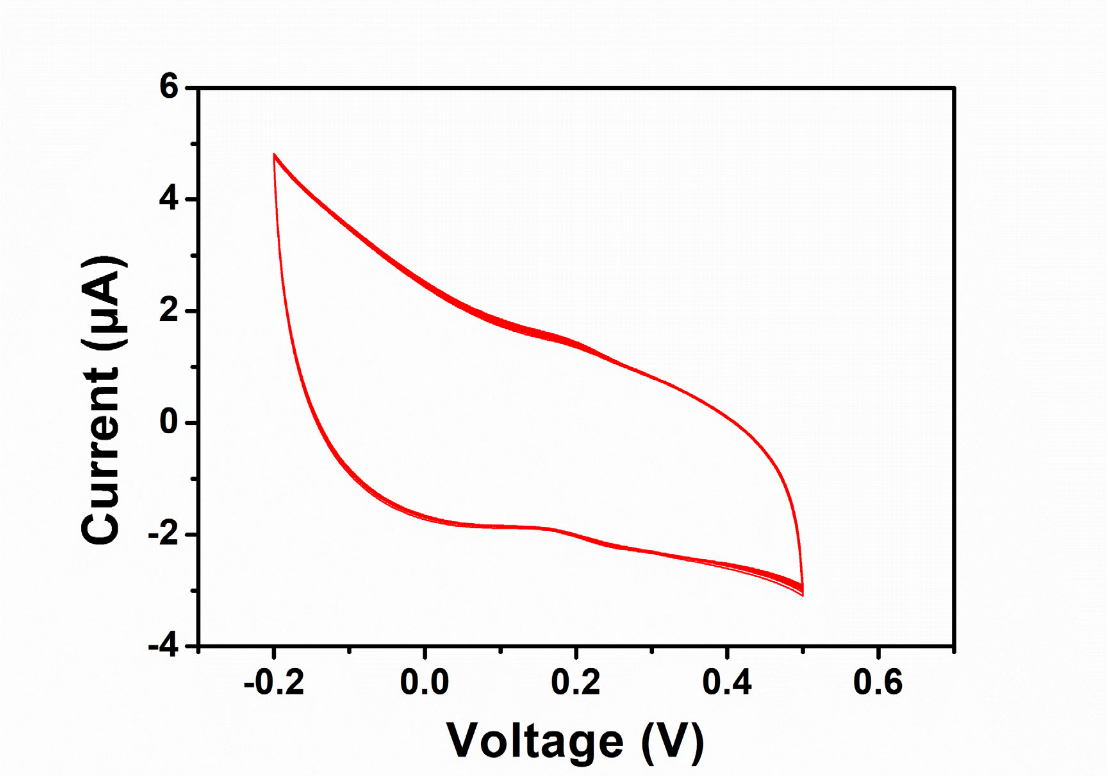

Figure S5. Cyclic voltammograms obtained by modified Pb^{2+}-aptasensor in 0.1 M PBS (pH 7.4) solution.

To explore the stability of the aptasensor, we added the aptasensors in 0.1 M PBS (pH 7.4) solution for 18 successive scanning cycles. As shown in Figure S5, the Cyclic voltammograms exhibited the excellent stability of the aptasensor.

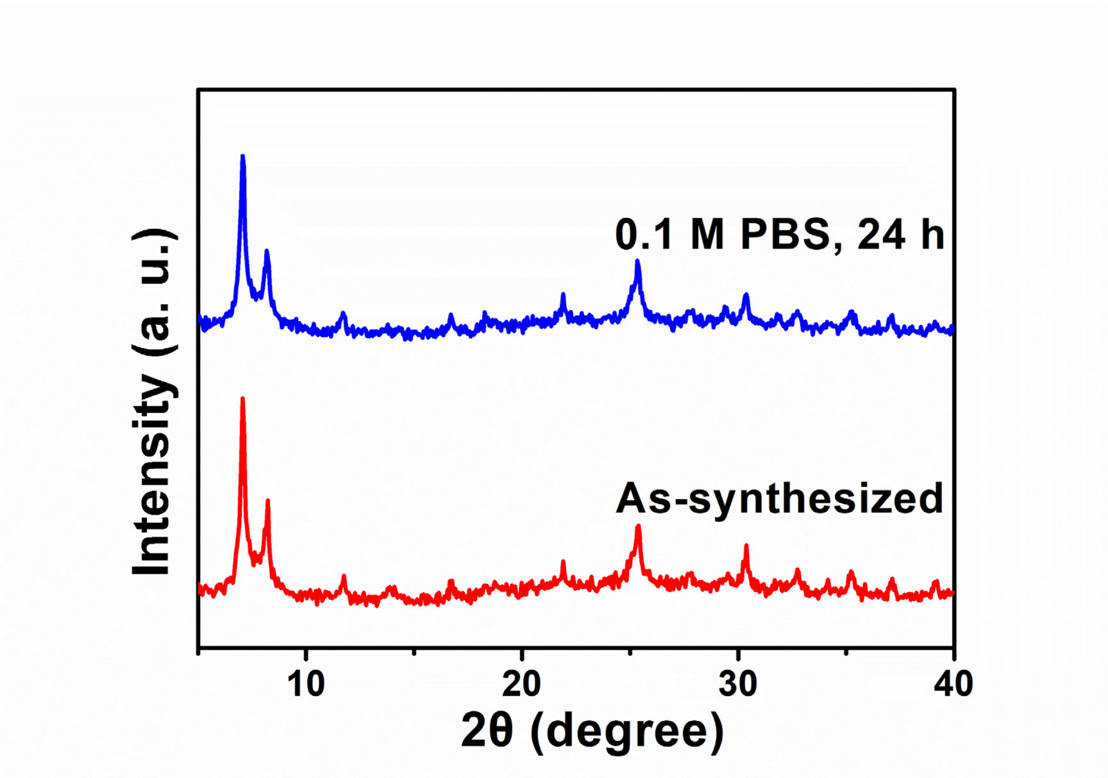

Figure S6. Powder X-ray diffraction patterns of MB@Zr-MOFs samples measured for chemical stability tests: as-synthesized sample and that soaked in 0.1 M PBS (pH 7.4) for up to 24 h.

The water stability of MB@Zr-MOFs was carefully explored by soaking the MB@Zr-MOFs in 0.1 M PBS (pH 7.4) solution and then tested by PXRD. The crystalline forms of the material are retained after being soaked in 0.1 M PBS (pH 7.4) solution for 24 h, which was far beyond the electrochemical testing time (Figure S5). The PXRD studies revealed that MB@Zr-MOFs soaked in water for 24 h showed identical PXRD patterns to their original samples, demonstrating that they can survive after water treatment.

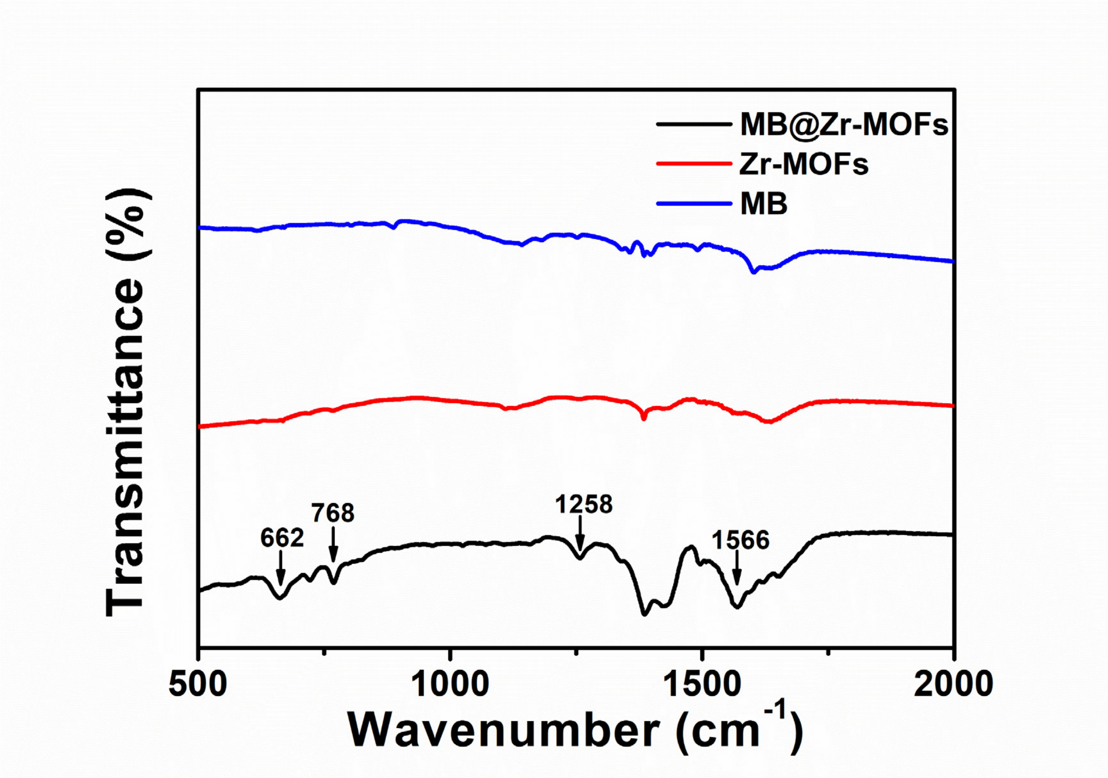

Figure S7. FT-IR spectroscopy of MB, Zr-MOFs and MB@Zr-MOFs.

Fourier transform infrared (FT-IR) spectroscopy was carried out to explore the variation. As shown in Figure S7, the characteristic peak at 1566 cm^{-1}, which was from the vibrations of C=C in the benzene ring, is greatly enhanced, demonstrating that the aromatic compounds increased. In addition, new characteristic peaks at 1258 cm^{-1}, 768 cm^{-1}, and 662 cm^{-1} were observed in MB@Zr-MOFs, indicating successful encapsulation of MB in Zr-MOFs.

Validation of the stepwise modification of SPEs

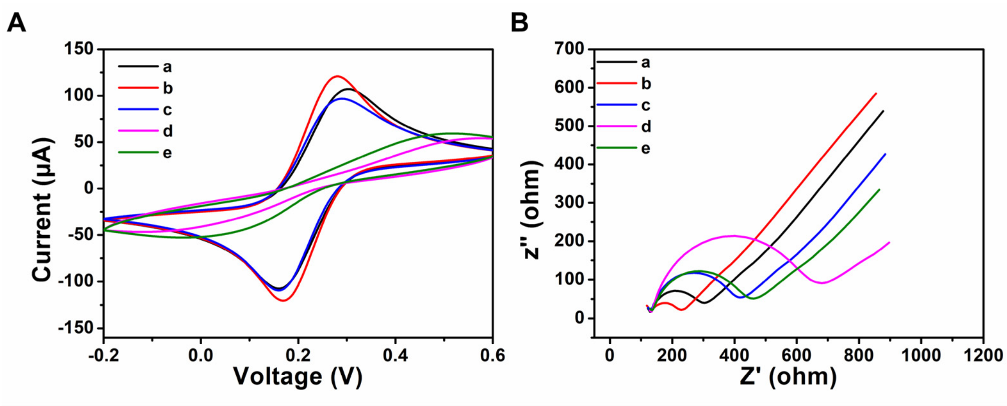

Figure S8. (A) CV and (B) EIS of the stepwise modifying validation of SPEs: (a) bare SPE, (b) AuNPs/SPE, (c) MB@Zr-MOFs-apt/AuNPs/SPE, (d) MCH/MB@Zr-MOFs-apt/AuNPs/SPE, (e) Cd^{2+}/MCH/MB@Zr-MOFs-apt/AuNPs/SPE. The concentration of Cd^{2+} is 10 ppb.

CV and EIS was employed to validate the successful assembly of each steps utilizing 5 mM K_3[Fe (CN)$_6$]/K_2[Fe (CN)$_6$] containing 0.1 M KCl in PBS (pH 7.4) buffer as the redox probe [1]. As shown in Figure S5, taken Cd^{2+} as an example, the bare SPEs exhibited symmetrical peak current (curve a). When AuNPs were modified on the SPEs, the peak current was high and symmetrical probably due to the good conductivity of AuNPs (curve b), which could accelerate the electron transfer in the redox pair reaction process [2]. The peak current decreased owing to the immobilization of the HMIs aptamers (curve c), which were negatively charged single strand DNA repelling the redox probe with same charge to reach the SPEs surface [3]. After MCH was incubated on the modified aptasensor, the peak current decreased on account of the block effect of MCH for redox probe to approach the electrode surface (curve d) [4]. Finally, the prepared aptasensing platform were cultured with HMI targets and showed enhancement of the peak current signals (curve e), demonstrating that HMIs-induced

aptamers conformational change enabled the electroactive redox probe close to the SPEs interface [4]. Therefore, the electron transfer of ferri/ferro cyanide was accelerated. Moreover, different modification steps were investigated by EIS (Figure S5). The bare SPE exhibited a small semicircle due to the low electron transfer resistance value of Ret (curve a) [5]. Then the Ret value decreased after dropped onto AuNPs (curve b), attributed to the excellent conductivity could effectively promote the electron transfer [2]. After incubated with MB@Zr-MOFs-apt, the semicicle of impedance increased owing to the self-assembly of the negatively charged aptamers obstructing anionic redox probe (curve c) [6]. Then MCH lead to the increase of semicircle owing to the surface passivation process (curve d) [6]. When Cd^{2+} was captured to the electrode, the impedance decreased, suggesting that Cd^{2+}-induced aptamer conformational change could shorten the distance between MB@Zr-MOFs to the SPE surface and then accelerate the electron transfer of the redox probe [4]. The obtained EIS results exhibited the same trend as CV, suggesting that the aptasensors were successfully modified stepwise.

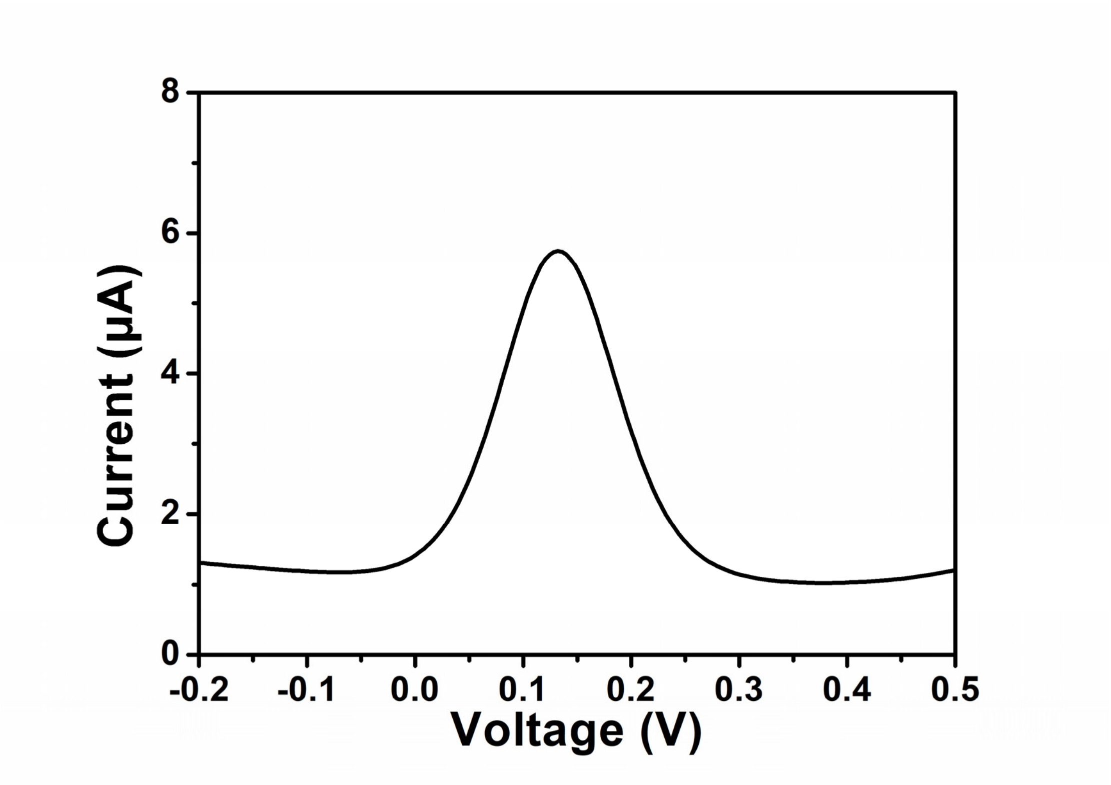

Figure S9. DPV curves of MCH/apt$_{Cd}$-MB@MOF/AuNPs/carbon rod.

Note-worthily, the current peak of MB@Zr-MOFs was located at about -0.23 V on gold electrode with a platinum wire as the counter electrode. The voltage window mainly depended on the selected electrodes. As shown in Figure S6, the DPV peak current of MB@Zr-MOFs was located at about +0.12 V on a carbon rod as a working electrode, which was in accordance with this work on SPEs at around +0.11 V.

Optimization of experimental conditions

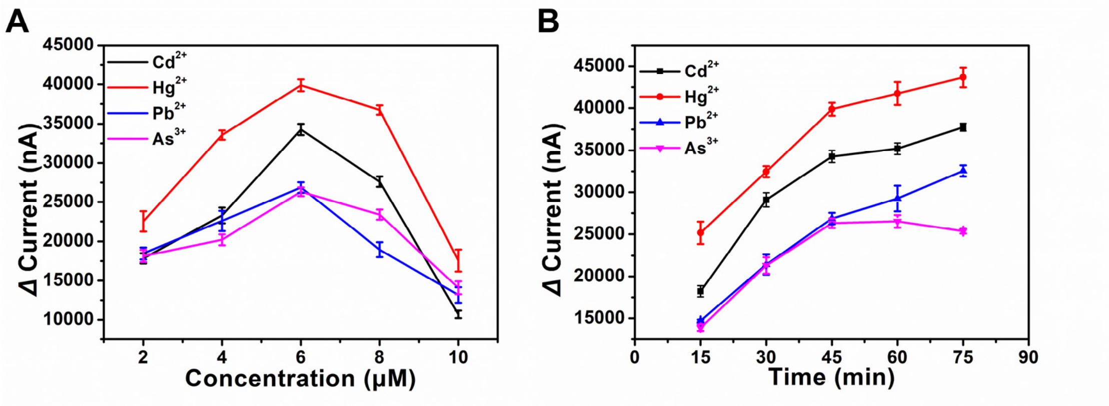

Figure S10. Optimizations of the (A) concentration of aptamers targeting Cd^{2+}, Hg^{2+}, Pb^{2+}, As^{3+} (B) incubation time of Cd^{2+}, Hg^{2+}, Pb^{2+}, As^{3+}.

Concentration of aptamers and incubation time of HMIs were considered as important factors to influence the sensitivity of the aptasensors. Therefore, the above mentioned two factors were optimized to achieve the satisfied current responses of the POCT electrochemical aptasensors.

The concentrations of aptamers varied from 2 to 10 μM with the gradient of 2 μM. As shown in Figure S7, The DPV *Δ*currents of four HMI aptasensors firstly increased until 6 μM owing to the increasing number of HMIs recognized and captured by corresponding aptamers to the SPEs surface. Afterward, the DPV *Δ*currents decreased probably due to the overcrowding effect of aptamers hindering the capture efficiency of HMIs targets, which might affect the folding of aptamer strand when recognized HMIs targets. Therefore, the optimal concentration of four aptamers were 6 μM.

Incubation time of HMIs was also estimated varied from 15 to 75 min with the gradient of 15 min. As shown in Figure S7, the DPV *Δ*currents of four HMI aptasensors were recorded in PBS buffer solution including 10 ppb of each HMIs. The current

signals increased until it reached a plateau at 45 min. Therefore, 45 min was considered as the optimal incubation time of HMIs.

Table S1. Comparison of analytical performance of POCT electrochemical platform for detection of HMIs with the previous reported HMIs detection methods.

Substrate	method	Range (ppb)				LOD (ppb)				Ref
		Cd^{2+}	Hg^{2+}	Pb^{2+}	As^{3+}	Cd^{2+}	Hg^{2+}	Pb^{2+}	As^{3+}	
GO, G-quadruplex	FL	-	0.1-10	-	-	-	0.034	-	-	**[7]**
SPCE-CB-AuNPs-Apt	SWV	1–50	-	-	-	0.14	-	-	-	**[1]**
BiNPs-SPE	LSV	0.1–150	-	-	-	3	-	-	-	**[8]**
GO-PB NPs	DPV	-	-	-	0.2–500	-	-	-	0.058	**[4]**
Stainless steel electrode	SWASV	-	20–1000	16–104	-	-	5.6	6.8	-	**[9]**
SPEs- AuNPs-MB@Zr-MOFs-Apt	DPV	0.2–20	0.1–10	0.1–10	0.1–20	0.039	0.039	0.073	0.022	**This work**

As shown in Table S1, the LODs of the proposed electrochemical POCT aptasensors have been measured to be 0.039 ppb for Cd^{2+}, 0.039 ppb for Hg^{2+}, 0.073 ppb for Pb^{2+}, 0.022 ppb for As^{3+}, which are competitive to those of previous individual detection methods based on FL (the LOD was 0.034 ppb for Hg^{2+}) [7], SWV (square

wave voltammetry) (the LODs was 0.14 ppb for Cd^{2+}) [1], LSV (linear sweep voltammetry) (the LOD was 3 ppb for Cd^{2+}) [8], and DPV (the LOD was 0.058 ppb for As^{3+}) [4]. The proposed electrochemical POCT platform also exhibited lower LODs and various kinds of HMIs than previous reported methods based on multiple detection of HMIs, including SWASV (square wave anodic stripping voltammetry) (the LODs were 5.6 ppb for Hg^{2+} and 6.8 ppb for Pb^{2+}) [9], indicating the ultrasensitivity and versatility of our proposed sensing platform. The low LODs in this work may probably due to the sufficient loading sites of MOF for MB to encapsulate, thus greatly amplifying the electrochemical signals of the aptasensors. Furthermore, most mentioned works rely on expensive and heavy detectors and are capable of detecting limited kinds of HMIs. The proposed electrochemical POCT platform not only realized low LOD of four HMIs, but also realizes rapid detection of four HMIs on a simple, cost-effective POCT aptasensing disk, which demonstrates that the proposed electrochemical POCT platform has good analytical performance and potential application prospects.

References

1. Fakude, C.T.; Arotiba, O.A.; Mabuba, N. Electrochemical aptasensing of cadmium (II) on a carbon black-gold nano-platform. *Journal of Electroanalytical Chemistry* **2020**, *858*, 113796, doi:https://doi.org/10.1016/j.jelechem.2019.113796.
2. Xiong, H.; Huang, Z.; Lin, Q.; Yang, B.; Yan, F.; Liu, B.; Chen, H.; Kong, J. Surface Plasmon Coupling Electrochemiluminescence Immunosensor Based on Polymer Dots and AuNPs for Ultrasensitive Detection of Pancreatic Cancer Exosomes. *Analytical Chemistry* **2022**, *94*, 837-846, doi:10.1021/acs.analchem.1c03535.
3. Xiong, H.; Gao, J.; Wang, Y.; Chen, Z.; Chen, M.-M.; Zhang, X.; Wang, S. Construction of an ultrasensitive electrochemiluminescent aptasensor for ractopamine detection. *Analyst* **2019**, *144*, 2550-2555, doi:10.1039/C9AN00183B.
4. Wen, S.-H.; Wang, Y.; Yuan, Y.-H.; Liang, R.-P.; Qiu, J.-D. Electrochemical sensor for arsenite detection using graphene oxide assisted generation of prussian blue nanoparticles as enhanced signal label. *Analytica Chimica Acta* **2018**, *1002*, 82-89, doi:https://doi.org/10.1016/j.aca.2017.11.057.
5. Zhang, J.-L.; Yang, Y.; Liang, W.-B.; Yao, L.-Y.; Yuan, R.; Xiao, D.-R. Highly Stable Covalent Organic Framework Nanosheets as a New Generation of Electrochemiluminescence Emitters for Ultrasensitive MicroRNA Detection. *Analytical Chemistry* **2021**, *93*, 3258-3265, doi:10.1021/acs.analchem.0c04931.
6. Kashefi-Kheyrabadi, L.; Kim, J.; Gwak, H.; Hyun, K.-A.; Bae, N.H.; Lee, S.J.; Jung, H.-I. A microfluidic electrochemical aptasensor for enrichment and detection of bisphenol A. *Biosensors and Bioelectronics* **2018**, *117*, 457-463, doi:https://doi.org/10.1016/j.bios.2018.06.036.
7. Guo, H.; Li, J.; Li, Y.; Wu, D.; Ma, H.; Wei, Q.; Du, B. A turn-on fluorescent sensor for Hg2+ detection based on graphene oxide and DNA aptamers. *New Journal of Chemistry* **2018**, *42*, 11147-11152, doi:10.1039/C8NJ01709C.
8. Fan, G. Highly Sensitive Electrochemical Determination of Cadmium (II) in Environmental Water Based on the Electrodeposited Bismuth Nanoparticles. *International Journal of Electrochemical Science* **2016**, *11*, 4362-4370, doi:10.20964/2016.06.5.
9. Kitte, S.A.; Li, S.; Nsabimana, A.; Gao, W.; Lai, J.; Liu, Z.; Xu, G. Stainless steel electrode for simultaneous stripping analysis of Cd(II), Pb(II), Cu(II) and Hg(II). *Talanta* **2019**, *191*, 485-490, doi:https://doi.org/10.1016/j.talanta.2018.08.066.
